# Self‐Produced Brain‐Like ECM From 3D‐Cultured Dermal Fibroblasts Enhances Neuronal Growth and Survival

**DOI:** 10.1002/biot.202400594

**Published:** 2025-03-10

**Authors:** Vincent Roy, Isabella Bienjonetti, Alexandre Paquet, François Gros‐Louis

**Affiliations:** ^1^ Department of Surgery Faculty of Medicine Laval University Quebec City QC Canada; ^2^ Division of Regenerative Medicine CHU de Quebec research Center Laval University Quebec City QC Canada

**Keywords:** 3D cell culture, brain‐like microenvironment, dermal fibroblast, extracellular matrix, neurogenesis, proteomic, tissue engineering

## Abstract

Studying neurological disorders in vitro remains challenging due to the complexity of the human brain and the limited availability of primary neural cells. Tissue engineering enables the development of three‐dimensional (3D) cell culture systems by generating a self‐produced extracellular matrix (ECM) substrate. Culturing cells within this ECM substrate is known to more effectively mimic physiological conditions compared to traditional monolayer cultures. In this study, we analyzed the proteome and matrisome of 3D cultured dermal fibroblasts embedded in a self‐produced ECM. Interestingly, in silico analysis predicted strong activation of neurogenesis‐associated functions in this tissue‐engineered 3D model. We showed that ECM proteins typically linked to neuronal development and maintenance were also expressed by dermal fibroblasts. Coculturing dermal fibroblasts with induced pluripotent stem cell (iPSC)‐derived motor neurons notably enabled long‐lasting culture periods while minimizing neuronal death, all without the need for costly media supplements. Furthermore, fibroblast‐conditioned media enhanced neuronal survival. Although we demonstrated that the dermal fibroblast‐derived ECM provided a rich matrix of proteins and signaling molecules that support neuronal growth and survival, the ECM alone seems insufficient to sustain the neuronal networks. These findings suggest that 3D cultured patient‐derived dermal fibroblasts generate a neuro‐supportive microenvironment and could serve as a cost‐effective and less invasive alternative to brain biopsies for modeling complex neurological disorders. This approach offers a promising platform for studying such neural growth and survival and exploring therapeutic strategies for neurological diseases.

## Graphical Abstract and Lay Summary

Studying neurological disorders in vitro remains challenging due to the complexity of the brain and limited availability of neural cells. This study demonstrated the potential of 3D tissue‐engineered in vitro model, made from human dermal fibroblasts embedded in a self‐produced brain‐like ECM, as a promising platform for supporting neuronal cells culture. The 3D cultured fibroblasts self‐produced ECM promoted neuronal growth and survival, providing a cost‐effective and less invasive alternative for modeling neurological disease.

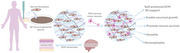

**Abbreviations**
2D: two‐dimensional
3D: three‐dimensional
ALS: amyotrophic lateral sclerosis
CNS: central nervous system
DMEM: Dulbecco–Vogt modification of Eagle's medium
ECM: extracellular matrix
FBS: fetal bovine serum
FC: fold change
GO: Gene Ontology
HPA: Human Protein Atlas
IPA: Ingenuity Pathway Analysis
iPSC: induced pluripotent stem cell
MN: motor neuron
NF1: Neurofibromatosis Type 1
NF‐M: neurofilament M
PBS: phosphate‐buffered saline
PCA: principal component analysis

## 1 Introduction

Neurological diseases encompass many complex and debilitating conditions affecting the central nervous system (CNS), often characterized by neuronal loss and functional deficits [1]. Despite significant research efforts, effective treatment options for many neurological diseases remain limited. Nowadays, different in vitro methods are used to study neurological diseases. However, in vitro human brain modeling is a complex approach that requires careful manipulation of neural cells and a deep understanding of the complex signaling pathways and interactions occurring in the CNS [2]. Although two‐dimensional (2D) cell culture has long been the standard, recent research has increasingly focused on three‐dimensional (3D) cell culture approaches that offer more realistic biochemical and biomechanical microenvironments. Indeed, neural cells obtained from diverse sources can be cultured in a 3D environment that mimics the structure and composition of the brain [3]. Such an environment can be achieved using various cell culture techniques and approaches, using scaffolding or hydrogels, which provide physical support for the cells to grow [4, 5]. 3D modeling offers many advantages over traditional 2D monolayer cultures, such as the simultaneous culture of different cell types, and the presence of cell‐to‐cell and cell‐to‐extracellular matrix (ECM) interactions [6, 7].

The ECM is a complex macromolecular network comprising fibrous proteins, such as collagens and polysaccharides. In the brain, the ECM fills the space between neurons and glial cells, and comprises about 20% of the total volume of the adult brain, whereas it can double in volume during development [8]. Beyond its structural role in the CNS, the ECM regulates various essential neural processes during brain development and contributes to both physiological and pathological conditions in the adult brain. These include cell migration, neurite outgrowth, differentiation, synaptogenesis, synaptic plasticity, and survival [9, 10]. Cells interact with core ECM components, such as collagens, fibronectin, laminins, and proteoglycans, via transmembrane receptors, among which integrins are the most prominent. These interactions result in transmitting various signals and modulating cellular behavior and survival [11, 12]. The core ECM undergoes dynamic remodeling, with changes in composition, organization, and stiffness [13]. In addition to core ECM components, ECM‐associated proteins play crucial roles in regulating cellular functions within the CNS. These proteins include various molecules such as ECM‐affiliated proteins, ECM‐remodeling enzymes, and secreted factors such as growth factors and cytokines. Those proteins contribute to the structural integrity of the ECM and participate in signaling pathways that govern neural development and function. Together, core‐ECM and ECM‐associated proteins form a complex network known as the “matrisome,” which orchestrates the dynamic interplay between neural cells and the microenvironment.

Besides a crucial role in normal development, alterations in the matrisome have been linked to various neurological diseases [14]. Disruptions in the composition, organization, or signaling properties of the matrisome have been implicated in a wide range of conditions, including neurodevelopmental disorders such as autism spectrum disorders, as well as neurodegenerative diseases like Alzheimer's disease, Parkinson's disease, and amyotrophic lateral sclerosis (ALS) [15]. Dysregulation of ECM remodeling enzymes, abnormal deposition of ECM proteins, or aberrant activation of ECM‐associated signaling pathways can harm neuronal connectivity, synaptic function, and overall brain homeostasis. Therefore, developing accurate in vitro models to study ECM dynamics in the context of neurological disorders is essential for identifying novel therapeutic targets and designing effective interventions to restore ECM homeostasis.

Although in vitro studies often utilize purified ECM proteins for cell culture coatings, there are not actually in vitro models to capture the full molecular complexity and heterogeneity of the brain's ECM [16]. However, reproducing the intricate complexity and diversity of the brain's ECM in vitro could significantly enhance the relevance and applicability of such studies. The tissue‐engineered skin model, made using the self‐assembly approach with patients’ own fibroblasts, has previously demonstrated to recapitulate specific neuropathological features of ALS [17] and Neurofibromatosis Type 1 (NF1) [18, 19]. During embryonic development, both the brain and the skin originate from the ectodermal germ layer [20, 21]. Although the brain and the skin differentiate into distinct structures, these two tissues could share common molecular signaling pathways and developmental processes. Understanding the intricate dynamics of the components of the matrisome is critical for elucidating the pathophysiology of neurological disorders. Hence, in the present study, we performed an in‐depth in silico analysis of the matrisome of 3D self‐assembled dermis. Through this analysis, we aimed to validate further the utility of scaffold‐free 3D tissue‐engineered models made of dermal fibroblasts and self‐produced ECM for studying neurological diseases in vitro. These data have the potential to provide insights into the underlying molecular and cellular mechanisms regulating neurogenic processes.

## 2 Materials and Methods

### 2.1 Human Fibroblast Isolation and Culture

Dermal fibroblasts were isolated from 6 mm skin punch biopsies taken from the same body region for each recruited individual as previously described [17]. Cells were cultured in Dulbecco–Vogt modification of Eagle's medium (DMEM; Invitrogen) supplemented with 10% fetal bovine serum (FBS; VWR), and an antibiotics cocktail comprising 100 IU/mL penicillin G (MilliporeSigma) and 25 µg/mL gentamicin (MilliporeSigma). Cells were maintained in an incubator at 37°C and 8% CO_2_ and media were changed three times per week. All experiments were conducted using fibroblasts at a maximum of five passages.

### 2.2 Tissue‐Engineered Dermis and Total Protein Extraction

3D dermis were produced using the self‐assembly approach of tissue‐engineering as previously described [18]. Briefly, 150,000 dermal fibroblasts were seeded in 6‐well plates and cultured in complete DMEM supplemented with 50 µg/mL ascorbic acid (MilliporeSigma) to enhance the secretion and assembly of ECM. Cells were cultured for 28 days in an incubator at 37°C, 8% CO_2_ and 95% relative humidity. Media was changed three times per week. 3D reconstructed dermis were snap frozen and then pulverized with a CryoMill apparatus (Retsch). For 2D cell culture, fibroblasts were maintained in a monolayer until a confluence of 70% was reached. Subsequently, cells were harvested, and proteins were extracted using TNG‐T buffer (50 mM Tris, pH 7.4, 150 mM NaCl, 10% glycerol, and 1% triton X‐100).

### 2.3 Conditioned Media and Total Secreted Protein Extraction

For both 2D and 3D cultured fibroblasts, cells were subjected to serum deprivation for 48 h before collecting supernatants. The conditioned media were centrifuged for 20 min at 2000 × *g* to remove cellular debris. Total protein was precipitated using the trichloroacetic‐sodium deoxycholate protein precipitation technique [22].

### 2.4 Nanoscale Liquid Chromatography Coupled to Tandem Mass Spectrometry

Total proteins were extracted from cells and conditioned media, collected by centrifugation, and washed with acetone. The pellets were solubilized in ammonium bicarbonate‐sodium deoxycholate buffer, and protein concentrations were measured using the Bradford assay (Bio‐Rad). Proteins were reduced with DTT, alkylated with iodoacetamide, and digested with trypsin overnight. The reaction was stopped with formic acid, and peptides were purified using a SOLAμ plate (Thermo Fisher Scientific) and then vacuum dried. The nanoLC‐MS/MS system containing a U3000 NanoRSLC liquid chromatograph (Thermo Fisher Scientific) in line with an Orbitrap Fusion Tribrid–ETD mass spectrometer (Thermo Fisher Scientific), driven by Orbitrap Fusion Tune Application (v. 3.3.2782.34), and equipped with a Nanospray Flex ion source was used to identify and quantify total proteins as previously detailed [23].

### 2.5 Bioinformatics

Raw data were processed with NetworkAnalyst 3.0 [24]. Low variance (15th percentile) and low abundance (5th percentile) proteins were filtered out. Then, data were normalized using a Log2 transformation and submitted to a Limma statistical analysis to evaluate the statistical significance of proteins regulation. Biological processes and tissue enrichment analysis were performed by inputting symbols of significantly regulated proteins into the Gene Ontology (GO) biological processes database via the EnrichR online tool [25], and DAVID enrichment analysis [26] using the Human Protein Atlas (HPA) normal tissue tool [27]. Additionally, in silico interactome and proteins, regulation predictions were generated and explored using Ingenuity Pathway Analysis (IPA) software (Qiagen) [28].

### 2.6 Differentiation of iPSC Into Motor Neurons

The induced pluripotent stem cells (iPSCs) were obtained from the iPSC‐Quebec platform of the CHU de Québec‐Université Laval Research Center. The iPSCs were reprogrammed from healthy human lymphoblastoid cells with the CytoTune‐2.0 Sendai Reprogramming Kit (Invitrogen) following the manufacturer's protocol. The differentiation of iPSCs into motor neurons (MNs) was conducted as previously described [29].

### 2.7 Culture of MNs in 3D Dermis

To culture MNs within the 3D fibroblast dermis,150,000 dermal fibroblasts were seeded in 12‐well plates and cultured in complete DMEM supplemented with 50 µg/mL ascorbic acid (MilliporeSigma). Paper anchors and weights were added to the culture plates, and cells were cultured for 21 days in an incubator at 37°C, 8% CO_2_, and 95% relative humidity. The media was changed three times per week. A total of 131,000 MNs/cm^2^ (at Day 18 of differentiation) were then seeded onto a dermal fibroblast sheet. MNs were cultured in 3D for 7 days with either MN differentiation medium, fibroblast medium (complete DMEM supplemented with 50 µg/mL ascorbic acid), or a 1:1 ratio of both media. A fibroblast sheet was then stacked on top of the 3D fibroblast dermis containing the MNs and cultured for 10 additional days under the same conditions (Figure 5A).

### 2.8 Immunofluorescence

MNs cultured in 2D on Day 18 of differentiation and within 3D dermis on Day 38 were fixed with 4% paraformaldehyde (Electron Microscopy Sciences) for 30 min at 4°C. Samples were blocked and permeabilized for 1 h at room temperature in a buffer containing phosphate‐buffered saline (PBS), 0.3% Triton X‐100 (Bio‐Rad), and 5% goat serum (Invitrogen). For 2D MNs, cells were incubated overnight at 4°C with specific primary antibodies against anti‐neurofilament M (NF‐M; 1:500; MilliporeSigma) and anti‐Islet‐1 (1:500; Abcam). For 3D cultures, only NF‐M (1:500; MilliporeSigma) was used. After washing, samples were incubated for 2 h with secondary antibodies: Alexa Fluor 594‐labeled donkey anti‐chicken (1:1000; Invitrogen) for NF‐M, Alexa Fluor 488‐labeled goat anti‐rabbit (1:1000; Invitrogen) for Islet1, and Hoechst 33342 (1:100; MilliporeSigma) for nuclear counterstaining. Images were captured using the LSI 700 confocal microscope with Zeiss Axio Imager (Carl Zeiss Microscopy).

### 2.9 Live/Dead Assay

At Day 11 of the differentiation protocol, 200,000 MNs/cm^2^ were plated on 12 well‐plates treated with 50 µg/mL poly‐d‐lysine (MilliporeSigma). Cells were cultured 7 days with MN medium according to the protocol described in Section 2.8. At Day 18 of differentiation, MNs were treated for 48 h with different ratios of conditioned medium collected from fibroblasts cultured in 3D and MN differentiation medium. The Live/Dead Viability/Cytotoxicity Kit for mammalian cells (Thermo Fisher Scientific) was used to assess viability and apoptosis as detailed by the manufacturer's procedure. Images were captured using confocal microscopy. The percentages of live and dead MNs were calculated with the ImageJ software. Ordinary one‐way analysis of variance (ANOVA) was performed using GraphPad Prism 10.2.3 software. The significance level was set a *p* value < 0.05.

## 3 Results

### 3.1 Differential Proteomic Landscape of Dermal Fibroblasts in 3D Culture

**FIGURE 1:**
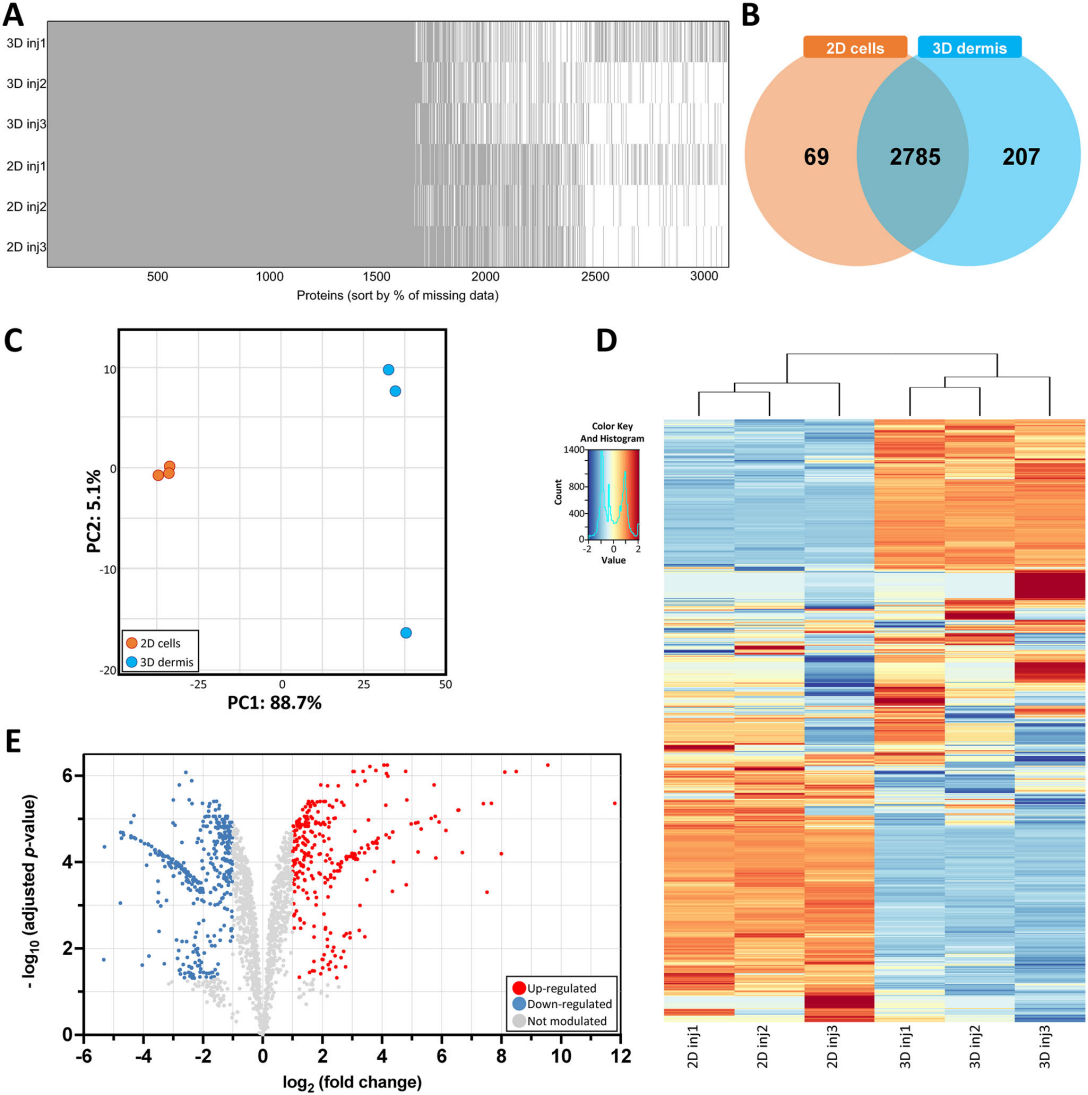
Comparative protein expression between 3D cultured and 2D monolayered fibroblasts. (A) The pattern of missing proteins in 2D and 3D fibroblast cultures, with three injections analyzed for each group. (B) Venn diagram of the identified proteins. (C) PCA illustrating the variance in protein expression between the culture approaches. (D) Proteomic heatmap profiles displaying protein intensity values across all samples, with hierarchical clustering of both rows and columns grouping samples with similar profiles. (E) Volcano plots showing the differentially expressed proteins in 3D cultured fibroblasts. 2D, two‐dimensional; 3D, three‐dimensional; PCA, principal component analysis.

We first conducted a proteomic analysis to identify differentially expressed proteins unique to the 3D context compared to standard 2D cultures. On average, 3059 proteins were identified, with missing values varying between 23.54% and 33%, resulting in an overall acceptable missingness rate of 28.03% for the dataset (Figure 1A). To address this, missing values were imputed with a noise value corresponding to the 0.01 percentile of the intensity distribution for each sample, enhancing data reliability as previously described [30]. Of the identified proteins, 2785 were shared between 2D monolayer and 3D fibroblast cultures (Figure 1B). A total of 69 proteins were exclusive to 2D culture cells, while 207 proteins were unique to 3D fibroblast cultures. Out of the identified proteins, 2206 showed sufficient expression levels for accurate quantification. Principal component analysis (PCA) and heatmap analysis confirmed the clear separation of samples by group (Figure 1C, D). PCA revealed that the first two principal components accounted for most of the variance, with PC1 explaining 88.1% and PC2 for 5.1%, highlighting the primary differences between the selected culture approaches (2D vs. 3D). Finally, the analyzed dataset comprised 1704 proteins that passed low variance and abundance filtering. Differential protein expression analysis, using an adjusted *p* value below 0.05 and a fold change (FC) equal or greater than 2 (Log2FC > 1), identified 298 significantly upregulated proteins and 368 significantly downregulated in 3D fibroblast cultures (Figure 1E, Table S1). Proteomic analysis of whole proteins collected from the conditioned media revealed similar trends (Figure S1). PCA clustering also demonstrated clear group separation, with PC1 explaining 74% of the variance attributed to the culture approaches (Figure S1A). Overall, 425 secreted proteins were quantified, with 70 upregulated and 141 downregulated proteins (Figure S1C and Table S2). A comparative analysis of proteins produced and released by the same cells identified 298 shared proteins, of which 30 showed increased expression and 8 showed decreased expression (Figure S1D). Collectively, these findings demonstrate that 3D culture significantly influences the protein expression of fibroblasts, as reflected both in cell‐associated proteins and those in conditioned media.

### 3.2 3D Cultured Fibroblasts Unveil Tissue‐Specific Protein Expression Associated With ECM Functions and Neurogenesis

**FIGURE 2:**
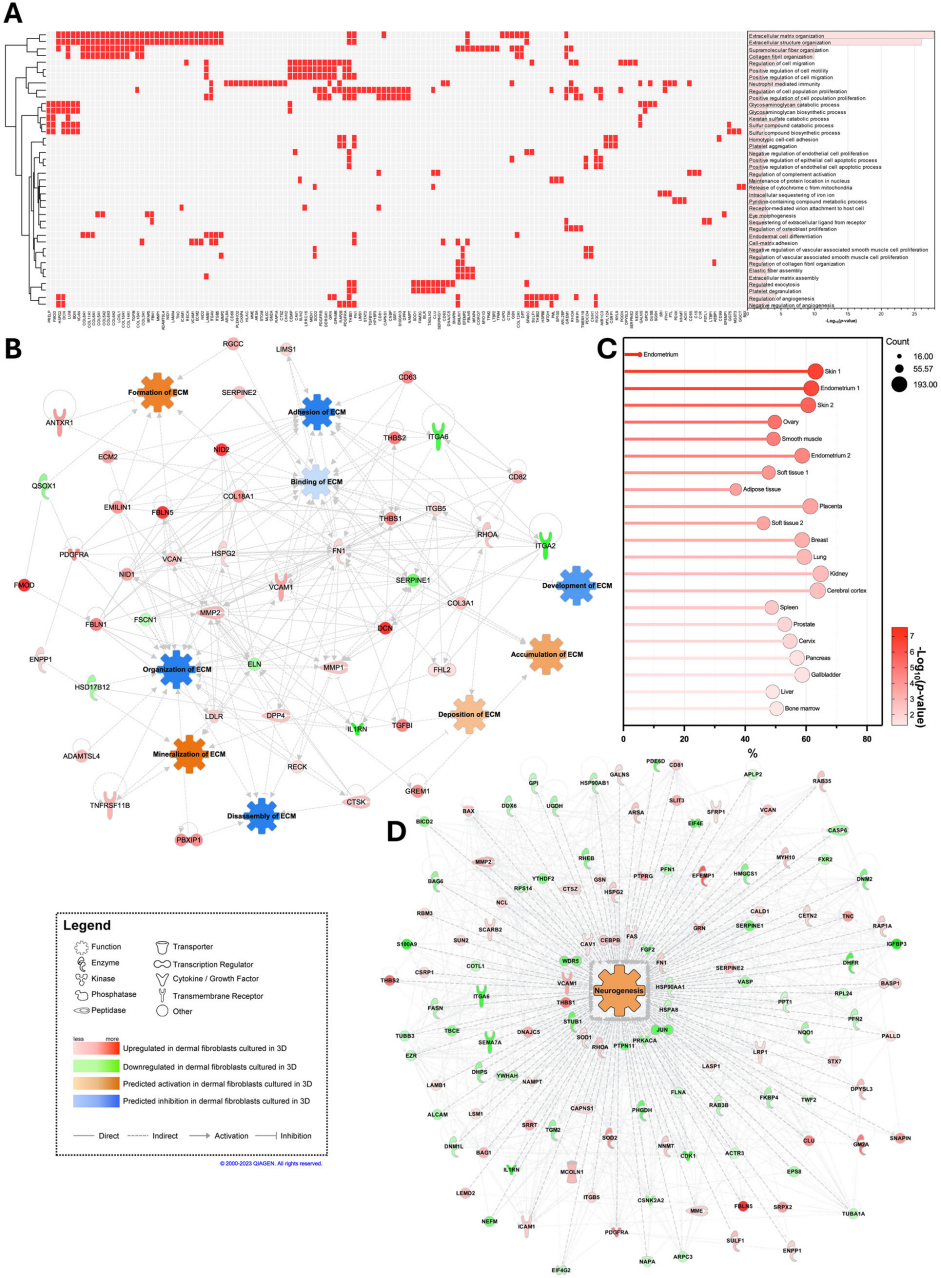
In silico analysis links protein content isolated from of 3D cultured fibroblasts to the matrisome and neurogenesis. (A) Clustergram of the top 40 enriched GO biological processes linked with upregulated proteins expressed by 3D cultured fibroblasts. (B) IPA‐generated interactome of significantly modulated proteins detected directly or indirectly implicated in ECM functions. (C) Tissue‐specific expression of upregulated proteins analyzed using the HPA normal tissue database. (D) IPA‐generated interactome of significantly modulated proteins. 3D, three‐dimensional; ECM, extracellular matrix; GO, Gene Ontology; HPA, Human Protein Atlas; IPA, Ingenuity Pathway Analysis.

To identify significantly enriched biological pathways associated with 3D fibroblasts culture, we performed a GO enrichment analysis on our dataset using only overexpressed proteins. The top 40 GO biological processes are shown in Figure 2A. Our analysis showed that upregulated proteins were strongly associated with the ECM. The top‐ranked biological process was *Extracellular matrix organization* and included many collagens. Other processes related to ECM organization, adhesion, assembly, biosynthesis, and catabolism were also linked with upregulated proteins. Additionally, other biological processes linked to cell proliferation and migration, as well as the regulation of angiogenesis were identified. To further understand the functional implications of the upregulated proteins, we use the IPA software to predict the activation or inhibition of various ECM functions using the entire differential protein expression dataset (Figure 2B). The protein expression profiles indicated the activation of ECM formation, accumulation, deposition, and mineralization, while predicting the inhibition of ECM adhesion, binding, development, organization, and disassembly. A GO enrichment analysis of biological processes with significant proteins shared with the secretome was conducted, also revealing numerous functions linked with the ECM (Figure S2).

The HPA normal tissue tool on the online DAVID bioinformatics platform was used to perform a tissue‐specific protein expression comparative analysis and localization [27]. This analysis revealed that the proteins, extracted from 3D cultured fibroblasts, were highly expressed in skin tissues (Skins 1 and 2) (Figure 2C). This comparative analysis also revealed that the expression levels of these proteins were also particularly prominent in other tissues, such as the endometrium, the ovary, the smooth muscle, and the soft tissue. Interestingly, the 3D cultured fibroblasts extracted proteins were also predominantly expressed in the brain with 190 of the 298 significantly upregulated proteins matching the cerebral cortex proteins (63.8%, *p* value = 0.002) (Figure 2C). Of particular interest, protein enrichment analysis using the IPA database revealed that the identified proteins (207 out of 298) were actively involved in the process of neurogenesis (GO:0022008 and GO:0050767) (Figure 2D). Note that these terms encompass all the processes involved in neurogenesis and could associated with various contexts such as development, tissue repair, proliferation, migration, differentiation, and maturation of new neurons. Broadly, this concept would therefore encompass the entire spectrum of processes involved in the maturation and functional establishment of neurons within the nervous system. Neurogenesis is a complex process coordinated by multiple functions, which few were further examined using the IPA software. Processes such as developmental process of synapse, synaptogenesis of neurons, axonogenesis, survival of neurons, and myelination were all predicted to be activated by differentially expressed proteins of 3D cultured fibroblasts (Figure S3).

### 3.3 In Silico Analysis Links Matrisomal Proteins of 3D Cultured Fibroblasts to Neurogenesis

**FIGURE 3:**
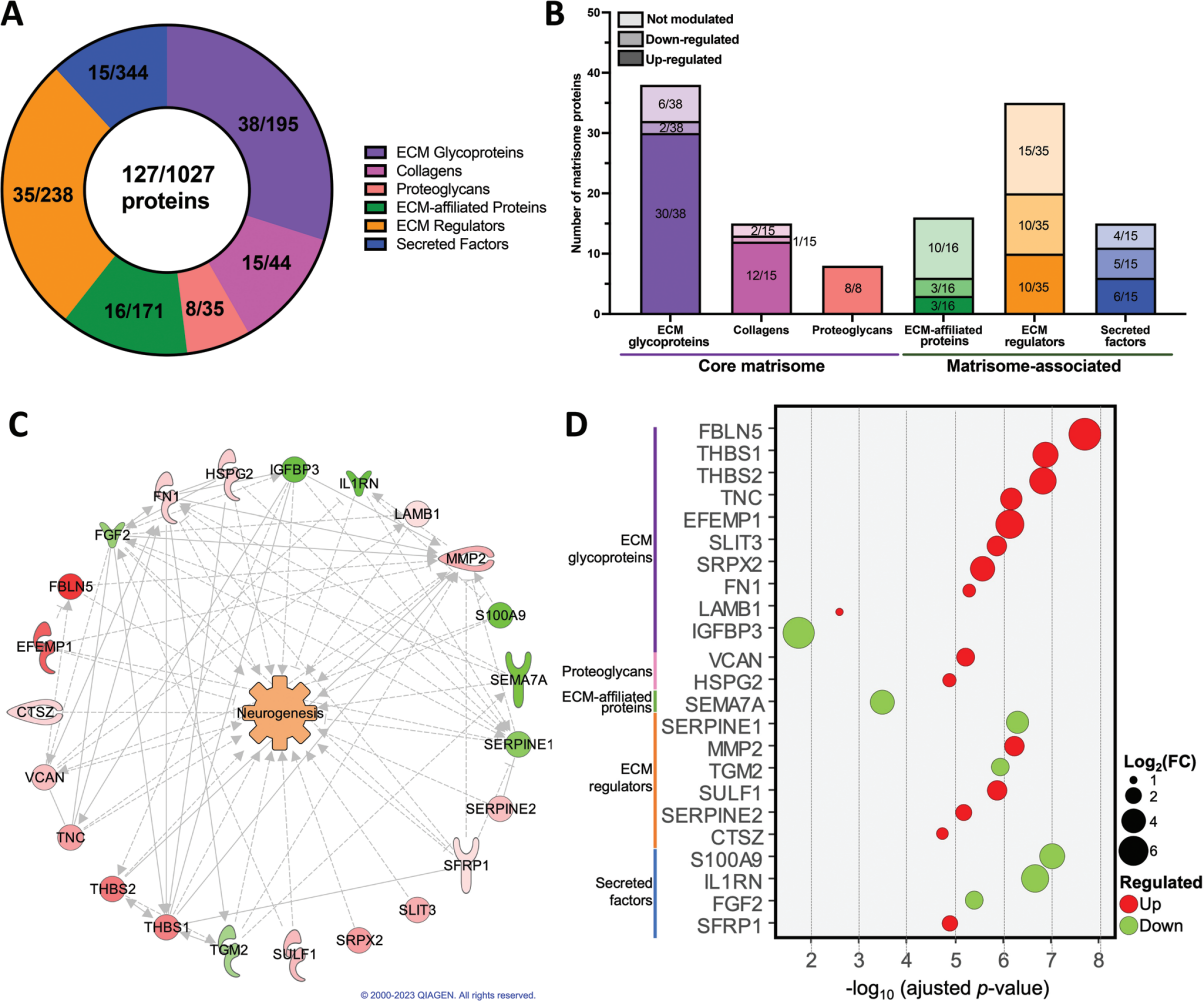
In silico analysis links matrisomal proteins of 3D cultured fibroblasts to neurogenesis. (A) Pie chart representation of the matrisome composition of 3D self‐assembled dermis compared to all human matrisome. (B) Bar chart illustrating the number of ECM proteins within each matrisome category that are upregulated, downregulated, or unchanged in 3D cultured fibroblasts compared to 2D monolayered fibroblasts. (C) IPA‐generated interactome of significantly modulated proteins detected in 3D cultured fibroblasts predicting neurogenesis activation. Refer to Figure 2 for the full‐color legend. (D) Dot plot of significantly modulated matrisomal proteins associated with neurogenesis. 2D, two‐dimensional; 3D, three‐dimensional; ECM, extracellular matrix; IPA, Ingenuity Pathway Analysis.

To further refine the analysis, we conducted a more focused examination of the matrisome protein content and the predicted in silico impact on neurogenesis. Based on the MatrisomeDB, we identified 127 quantified proteins considered part of the core ECM and ECM‐associated human proteins (Figure 3A and Table S3). Among these, we identified 38 glycoproteins, 15 types of collagens, and 8 proteoglycans, all categorized as core matrisome proteins. Additionally, 3D cultured fibroblasts expressed 16 ECM‐affiliated proteins, 35 regulators, and 15 secreted factors, which fall under the category of matrisome‐associated proteins.

We then examined the proper modulation of each ECM proteins in both culture approaches (3D vs. 2D). Overall, the majority of the quantified core matrisome proteins were significantly upregulated in 3D cultured fibroblasts, while greater variability in ECM‐associated proteins expression was observed (Figure 3B). Matrisome‐associated protein enrichment analysis also identified, using the IPA prediction tool, 23 ECM proteins directly or indirectly involved in the positive regulation of neurogenesis (Figure 3C, D).

### 3.4 Comparative Analysis of 3D Cultured Fibroblast Matrisome With Human Brain Proteomic Studies

**FIGURE 4:**
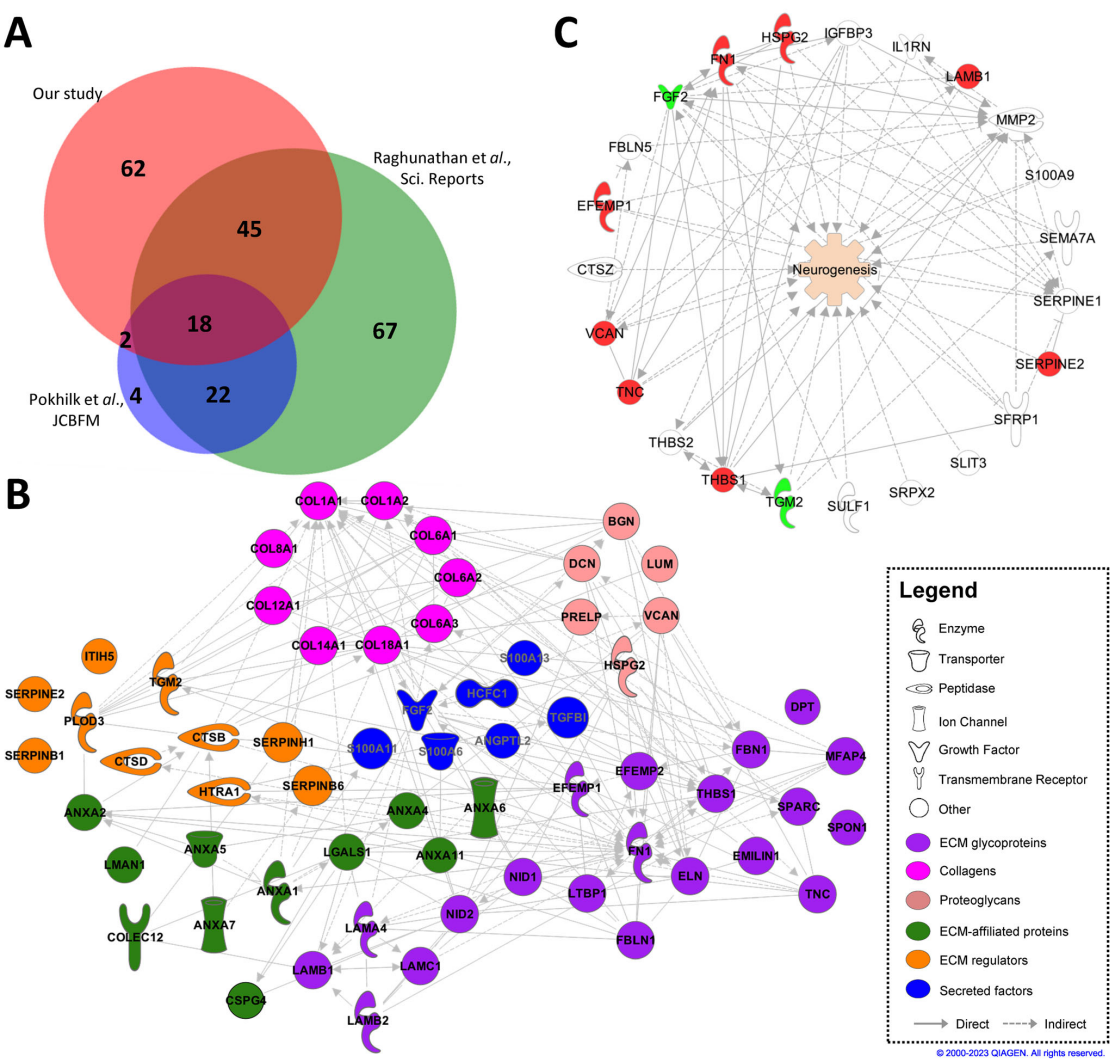
Comparative analysis with publicly available brain matrisome databases. (A) Venn diagram of matrisomal and total proteins shared with data extracted from Raghunathan et al. and Pokhilko et al. (B) Interactome of the matrisomal proteins shared between our data and Raghunathan et al. dataset clustered by matrisome categories. (C) In silico analysis of neurogenesis using only common matrisomal proteins.

We next sought to validate our findings by comparing our dataset with previously published studies reporting proteomic analyses on human brain tissues [31, 32]. In these studies, Raghunathan et al. reported the identification of a total of 4395 proteins in postmortem brains obtained from both Parkinson's disease patients and healthy individuals, whilst Pokhilko et al. identified a lower number of proteins with 1524 (Figure 4A). We then compared our dataset, composed of 2206 quantified proteins, with those previously published from both of these studies, examining only the presence or absence of proteins without comparing absolute expression values. Notably, we identified 710 proteins that were commonly shared between all datasets (Figure 4A), with a 71.2% concordance between our dataset and the Raghunathan's data (shared proteins: 1571), whilst the overlap with the Pokhilko's data was merely 33.8% (shared proteins: 746).

With the aim of identifying shared ECM proteins, we then compared MatrisomeDB‐associated proteins between these studies. Interestingly, 63 ECM proteins were found to be commonly detected with the Raghunathan's dataset (Figure 4A). Specifically, these 63 shared matrisome‐associated proteins were composed of several glycoproteins (31.7%), collagens (12.7%), proteoglycans (9.5%), and numerous ECM‐associated proteins and regulators (33.3%) (Figure 4B). Interestingly, our in silico analysis, focusing on brain matrisome proteins detected within our 3D model, predicted a significant activation of neurogenesis. Remarkably, among the 63 commonly detected brain matrisome proteins, the differential expression of certain key proteins appeared sufficient to drive this neurogenic response in silico (Figure 4C). This highlights the potential role of the collective ECM protein network in promoting neurogenesis.

### 3.5 Dermal Fibroblasts Cultured in 3D Promote Axon Formation and Neurons Survival In Vitro

**FIGURE 5:**
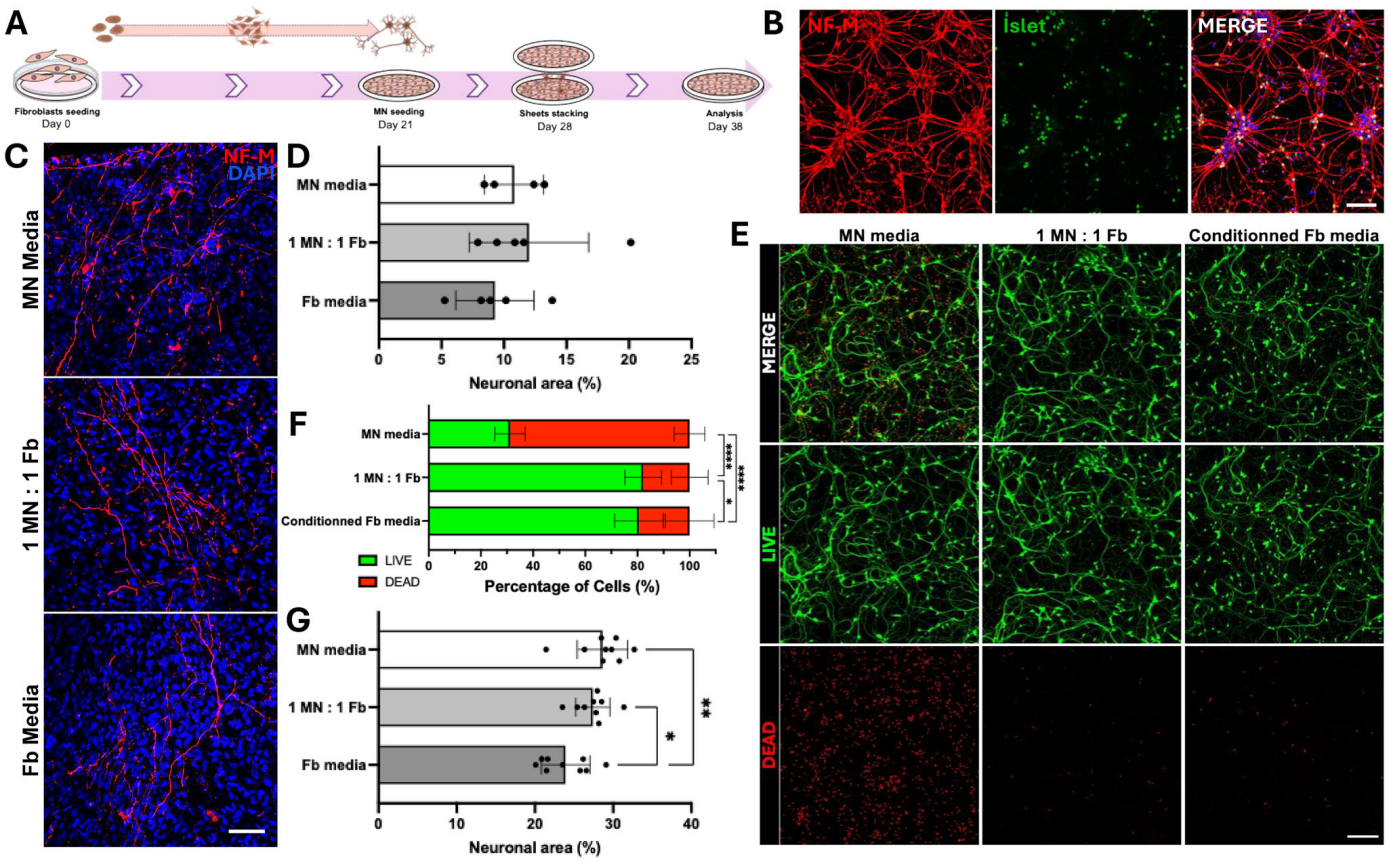
Dermal fibroblasts cultured in 3D create a scaffold supporting neuron survival and axon formation. (A) Schematic representation of the protocol for coculturing iPSC‐derived MN within self‐produced ECM by 3D cultured fibroblasts. (B) Immunofluorescence of specific neuronal markers on Day 18 in iPSC‐derived MN cultured in 2D. (C) Immunofluorescence of NF‐M (red) in iPSC‐derived MN cultured in self‐produced ECM by 3D cultured fibroblasts using different media ratios (MN, 1:1 MN:Fb or Fb). (D) Averaged area covered by seeded MNs incorporated into the 3D self‐produced ECM. (E) Live–Dead assay of MN treated for 48 h with MN medium, 3D fibroblasts‐conditioned media, or a 1:1 mixture. (F) Percentage of living and dead cells cultured with different ratios of MN and 3D fibroblast‐conditioned. Scale bar = 100 µm. (G) Average area occupied by viable MNs cultured within the 3D self‐produced ECM using different culture media. A one‐way ANOVA with Tukey's multiple comparison test were performed. Graphs show mean ± SD. *N* = 3, *n* = 3. * *p* < 0.05, ** *p* < 0.01 and **** *p* < 0.0001. 2D, two‐dimensional; 3D, three‐dimensional; ANOVA, analysis of variance; ECM, extracellular matrix; iPSC, induced pluripotent stem cell; MN, motor neuron; NF‐M, neurofilament M; SD, standard deviation.

To validate our in silico prediction, MNs derived from iPSC were incorporated into the tissue‐engineered dermis as illustrated (Figure 5A). The iPSC differentiation into mature MNs was validated by immunofluorescence using specific markers such as NF‐M and ISLET (Figure 5B). The formation of neural axons within the 3D scaffold generated by dermal fibroblasts was subsequently assessed (Figure 5C). Axons were well‐organized when the model was cultured in a medium composed of an equal ratio of fibroblasts and neurons media, compared to other conditions. In the condition with only fibroblasts media, fewer neurons were observed, while the axonal network appeared more disorganized in the condition using neurons media alone. Although the neuronal morphology appeared to be influenced by the media, no significant difference was observed in the area occupied by the MNs (Figure 5D). A Live–Dead assay on MNs demonstrated that exposure to conditioned media derived from 3D dermal fibroblast cultures, or a 1:1 mixture of this conditioned media with MNs culture media, improved neurons survival (Figure 5E, F). Interestingly, significantly fewer living MNs were observed when the cells were exposed exclusively to fibroblast‐conditioned media (Figure 5G). These results therefore suggest that the molecules secreted by fibroblasts contributed to neurogenesis and neuronal survival, but there were not sufficient to sustain the efficient culture of MNs in vitro. Furthermore, in silico prediction indicated that the secretome of 3D cultured fibroblasts is likely to activate processes associated to cell survival, including neural cells (Figure S4).

## 4 Discussion

In recent years, complex 3D cell culture‐generated models have gained popularity over traditional 2D cultures and in vivo models [7]. Despite progress, a significant gap exists in the availability of in vitro models that accurately replicate the brain's ECM complexity and composition, which is critical for understanding the role of the ECM in neurological processes. The present study aimed to evaluate the utility of 3D cultured dermal fibroblasts as an exogen‐free and self‐produced ECM scaffold for investigating neurobiological processes. Our findings demonstrated that human dermal fibroblasts displayed distinct protein expression profiles intrinsic to the selected cell culture 3D cell culture approach. Specifically, the 3D tissue‐engineered model allowed primary human dermal fibroblasts to secrete and assemble a complex ECM [33]. Actually, mass spectrometry analysis showed significant enrichment of ECM proteins and ECM‐associated proteins in 3D cultured fibroblasts compared to fibroblasts grown in monolayered cultures. Notably, our in‐depth comparative analyses revealed strong similarities between the matrisome of 3D cultured fibroblasts and the human brain. Furthermore, we identified several ECM proteins within our dataset that are also predominantly expressed in both skin tissues and the human brain, underscoring this model's potential relevance. Interestingly, recent studies have demonstrated that tissue‐specific ECM can significantly enhance the physiological relevance of in vitro models [16]. Additionally, this approach accelerates the formation and maturation of neuronal networks by promoting earlier synchronized neural activity and improving network organization.

The human skin and brain share many ECM components, including collagens, fibronectin, laminins, and proteoglycans [34, 35]. Of particular interest, we identified core matrisomal proteins and matrisome‐associated proteins shared with the 3D fibroblast dermis and the human brain, which are predicted in silico to promote neurogenesis. Therefore, neurogenesis‐related processes, such as synaptogenesis, axonogenesis, myelination, and neuron survival, were also predicted to be activated in our model. This suggests that proteins expressed by 3D dermal fibroblasts create a proneurogenic microenvironment enabling neuron cell culture and the formation of a functional neuronal network. Among the neurogenic proteins identified in 3D dermis, several matrisomal proteins were differentially expressed, many of which are also found in the human brain. These proteins are known to play essential roles in the development and repair of the nervous systems, and in preserving neural function during injury or disease [11]. Although some proteins displayed significant differential expression, the contribution of these proteins to neurogenesis likely arises from the collective influence of the broader ECM protein network rather than the isolated effects of individual components. This underscores the importance of considering the holistic interactions within the ECM when studying neurogenic processes. Such interactions are particularly relevant in the context of the brain, where the ECM forms a complex and dynamic network of proteins and other molecules providing structural support, regulating cell behavior, and influencing neural development and function [10]. The ECM acts as a scaffold for neural stem cells, facilitating the activation and proliferation of these cells through mechanical cues and delivery of crucial growth factors and signaling molecules [36]. Additionally, the ECM plays a pivotal role in the formation and stabilization of synapses between newly generated neurons and their targets, promoting the establishment of functional connections within neurogenic niches [37]. Building on this understanding, recent research highlighted the critical role of ECM components in the brain and other tissues, such as the skin, where fibroblasts serve as key contributors to ECM production and organization [16, 38]. Although the brain ECM is distinct in composition, being enriched with unique proteoglycans such as brevican, neurocan, and tenascin‐R, studies have shown that ECM derived from fibroblasts can mimic several functional aspects of brain ECM as well as replicate established pathological features while culturing patient‐derived fibroblasts into a self‐produced ECM [17, 18, 19]. Previous work by Lam et al. demonstrated incorporating ECM substrates into neuronal cultures accelerates network formation and maturation [16]. Indeed, tissue‐specific ECM has been shown to be critical in enhancing neuronal health and functionality, particularly in long‐term cultures. For instance, MaxGel, a commercially available human ECM cocktail derived from skin cell cultures, has been demonstrated to support neuronal cultures as effectively as brain‐specific ECM [16]. These advancements underscore the potential of fibroblast‐based systems to bridge the gap between simplistic in vitro models and the complex microenvironment of the brain, making them valuable tools for investigating CNS development, regeneration, and disease.

Cultivating neurons in 3D using standard methods remains a significant challenge, often being labor‐intensive, time‐consuming, and difficult to optimize. Our findings demonstrated that dermis reconstructed from 3D cultured fibroblasts not only processes an ECM comparable to that of the CNS but also creates a proneurogenic environment supporting neuronal culture and axon formation. Emerging evidence suggests the critical role of CNS fibroblasts in supporting key functions of neuronal progenitor cells, including proliferation and migration [38]. Moreover, the connection between fibroblasts and the brain is increasingly acknowledged, particularly in networked communication through signaling pathways, neuroimmune interactions, and paracrine secretion of neurotrophic molecules [39]. Building on these findings, our study revealed that iPSC‐derived MNs can be successfully cocultured with skin fibroblasts, enabling survival over an extended culture period. This coculture system offers a robust platform for investigating neurodegenerative diseases in vitro, combining physiological relevance with practical scalability, and holds great potential for advancing personalized medicine by enabling patient‐specific studies and tailored therapeutic strategies. A key advantage of this approach lies in the fibroblast‐derived self‐secreted ECM, which partially replicates the CNS microenvironment. This ECM provides a supportive matrix for neuronal cultures without requiring media supplementation with costly exogenous additives, making the ECM particularly advantageous for neuroscience and stem cell research. Our results demonstrated that the fibroblast‐derived ECM and the secreted factors within conditioned media promoted neuronal outgrowth and survival, creating a proneurogenic environment. However, these components alone are insufficient to sustain the neuronal network over time. The neurons are also likely to contribute to this environment by secreting their own ECM proteins, which may enhance neural activity in vitro and compensate for ECM components that dermal fibroblasts cannot produce [40]. This interplay between fibroblast‐derived and neuron‐secreted ECM highlights the dynamic nature of this system and underscores the potential of this system as a versatile and physiologically relevant platform for studying neurobiological processes and therapeutic development.

Each 3D model has unique strengths and limitations, with the choice depending on the specific research questions. In the nervous system, the ECM is highly dynamic, supporting processes such as neural development, synaptic plasticity, neuronal survival, and injury repair [40]. Dysregulation of ECM turnover, which involves continuous synthesis and proteolysis, can contribute to disease progression [12, 14]. Capturing these rapid ECM dynamics is challenging in animal models, giving tissue‐engineered systems a distinct advantage for studying ECM‐related disorders.

Our model offers several key advantages over other 3D systems. The dermal fibroblast‐derived ECM closely mimics the natural extracellular environment, providing a rich matrix of proteins and signaling molecules that support neurogenesis and neuronal survival. Additionally, the described 3D culture system replicates the structural complexity of in vivo environments while maintaining simplicity, reproducibility, and control. Dermal fibroblasts are easily accessible peripheral cells that can be obtained noninvasively from patients, efficiently reprogrammed into neuronal cells, and biobankable, making them a scalable, cost‐effective, and practical model for a wide range of applications [41]. Finally, the model is highly customizable, allowing integration with other cell types to study cell‐ECM interactions under diverse conditions. These features position our model as a versatile and efficient tool for exploring neurobiology, ECM‐specific mechanisms and pathways, and receptor–ligand interactions. Although our model offers advantages over others, we acknowledge that factors beyond fibroblast secretions, such as the serum components, may also contribute to the observed effects. Building on the strengths of tissue‐engineered models, the emergence of high throughput screening techniques and advanced analysis tools in the “omics” era, such as quantitative mass spectrometry, has further transformed the study of complex biomolecular dynamics. These innovations are increasingly applied to neurological disease research, enabling the investigation of the molecular complexity and the underlying pathogenic mechanisms that remain poorly understood [42]. Indeed, mass spectrometry has provided unprecedented insights into specific protein expression profiles of various regions of the human brain. Integrating these proteomics datasets with open‐source databases derived from novel in vitro studies, including personalized systems using patients‐derived fibroblasts, represents the next step in advancing the understanding of neurological disorders.

## 5 Conclusion

Gaining a comprehensive understanding of the intricate interactions between the ECM and neural cells will be crucial for elucidating the underlying mechanisms involved in neurological diseases and for developing therapeutics. This highlights the importance of maintaining the native molecular complexity of the ECM, as it plays a critical role in regulating neuronal growth, neurogenesis, and survival. Although our model utilizes dermal fibroblast‐derived ECM, it demonstrates a comparable capacity to provide an ECM‐rich microenvironment that supports neurogenesis. The substantial molecular similarity between the matrisome of our 3D cultured fibroblasts and the brain ECM further supports the utility of this model for studying CNS processes.

These findings collectively emphasize the value of 3D dermal fibroblast‐based models as a scalable, cost‐effective, and physiologically relevant alternative for investigating neurological diseases. Although our results indicate that the fibroblast‐derived ECM provides an enriched microenvironment for neurogenesis, future studies could explore integrating tissue‐specific ECM or enhancing the fibroblast‐derived ECM to further improve its mimicry of CNS‐specific characteristics. These efforts would advance the development of more refined models that bridge the gap between in vitro studies and in vivo complexities. In this regard, culturing patient‐derived dermal fibroblasts in 3D cultures could offer a valuable and less invasive alternative to brain biopsies, providing in vitro personalized models to study complex neurological disorders.

## Author Contributions

Conceptualization: Vincent Roy and François Gros‐Louis. Methodology: Vincent Roy and Isabella Bienjonetti. Formal analysis: Vincent Roy, Isabella Bienjonetti, and Alexandre Paquet. Writing–original draft preparation: Vincent Roy, and François Gros‐Louis. Writing–review and editing: Alexandre Paquet and Isabella Bienjonetti. Supervision: François Gros‐Louis. Funding acquisition: François Gros‐Louis. All authors have read and agreed to the published version of the manuscript.

## Ethics Statement

Healthy individuals were enrolled on a voluntary basis and signed a consent form approved by our Institutional Ethics Committees (Protocol number: 2012–1316). All experiments were made in agreement with the national Tri‐Council Policy Guidelines: Ethical Conduct for Research Involving Humans and approved by the ethics committee of the CHU de Quebec–Université Laval. For more information, please contact gurecherche@chuq.qc.ca.

## Conflicts of Interest

The authors declare no conflicts of interest.

## Supporting information



Supporting Information

Supporting Information

Supporting Information

Supporting Information

## Data Availability

The data supporting the findings of this study are provided in the Supporting Information of this manuscript, while the raw data are available upon request.
