# Supplementary material for: Self‐Produced Brain‐Like ECM From 3D‐Cultured Dermal Fibroblasts Enhances Neuronal Growth and Survival
Source: Biotechnol J. 2025 Mar 10;20(3):e202400594. doi: 10.1002/biot.202400594 (PMC11891511; doi:10.1002/biot.202400594)
Supplement: Supplementary file 1 — Supporting Information [file BIOT-20-e202400594-s001.docx]

**Supplementary Figures**

**Figure S1 – Comparison of the secreted proteins profiles of 3D self-assembled dermis to those of standard 2D fibroblasts and the composition of 3D dermis.** **(A)** PCA illustrating the variance in protein secretion between the 3D and 2D fibroblast cultures. **(B)** Proteomic profiles heatmap representing the intensity values in all samples of all secreted proteins of the analysis. Both rows and columns were clustered hierarchically to display similar samples or proteins with similar profiles close to each other. **(C)** Volcano plots showing the differentially expressed proteins secreted by 3D dermis, with 70 proteins significantly up-regulated and 141 proteins significantly down-regulated. **(D)** Venn diagram illustrating the proteins identified in 3D self-assembled dermis and its conditioned media, with a comparison of the up- and down-regulated shared proteins.

**Figure S2 – Clustergram of enriched GO biological processes associated with proteins significantly oversecreted by dermal fibroblasts cultured in 3D.** The column represents the input proteins, and the matrices indicate the top 10 enriched GO terms associated with these proteins.

**Figure S3 – IPA-generated interactome of functions related with neurogenesis.** Significantly expressed proteins of 3D dermis were directly or indirectly connected to functions such developmental process of synapse, synaptogenesis of neurons, axonogenesis, myelination and cell survival of neurons, which were all predicted to be activated *in silico*. The legend is in Figure 2.

**Figure S4 – IPA-generated interactome of cell survival promoted by the secretome.** Significantly secreted proteins quantified in conditioned media of 3D dermis were directly or indirectly connected to cell survival and survival of neural cells, which were all predicted to be activated *in silico*. The legend is in Figure 2.
